# Advances and challenges in drug design against dental caries: Application of *in silico* approaches

**DOI:** 10.1016/j.jpha.2024.101161

**Published:** 2024-12-09

**Authors:** Zhongxin Chen, Xinyao Zhao, Hanyu Zheng, Yufei Wang, Linglin Zhang

**Affiliations:** a State Key Laboratory of Oral Diseases & National Center for Stomatology & National Clinical Research Center for Oral Diseases, West China Hospital of Stomatology, Sichuan University, Chengdu, 610041, China; b Department of Endodontic and Operative Dentistry, West China Hospital of Stomatology, Sichuan University, Chengdu, 610041, China; c The College of Life Science, Sichuan University, Chengdu, 610041, China

**Keywords:** Dental caries, *Streptococcus mutans*, Computer-aided drug design, AI-Assisted drug design, Glucosyltransferases, Antigen I/II, Sortase A

## Abstract

Dental caries, a chronic disease characterized by tooth decay, occupies the second position in terms of disease burden and is primarily caused by cariogenic bacteria, especially *Streptococcus mutans,* because of its acidogenic, aciduric, and biofilm-forming capabilities. Developing novel targeted anti-virulence agents is always a focal point in caries control to overcome the limitations of conventional anti-virulence agents. The current study represents an up-to-date review of *in silico* approaches of drug design against dental caries, which have emerged more and more powerful complementary to biochemical attempts. Firstly, we categorize the *in silico* approaches into computer-aided drug design (CADD) and AI-assisted drug design (AIDD) and highlight the specific methods and models they contain respectively. Subsequently, we detail the design of anti-virulence drugs targeting single or multiple cariogenic virulence targets of *S. mutans*, such as glucosyltransferases (Gtfs), antigen I/II (AgI/II), sortase A (SrtA), the VicRK signal transduction system and superoxide dismutases (SODs). Finally, we outline the current opportunities and challenges encountered in this field to aid future endeavors and applications of CADD and AIDD in anti-virulence drug design.

## Highlights

• Specific methods and models with CADD and AIDD are comprehensively summarized.
• The design of single- and multi-target drugs against *Streptococcus mutans* are detailed.
• The current limitations of targeted anti-caries drug design were discussed.

## Graphical abstract

**Figure:**
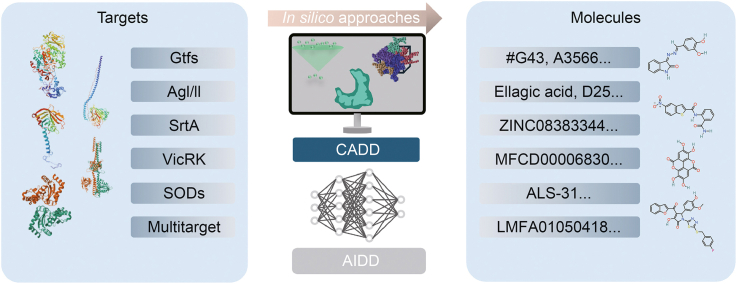

## 1 Introduction

Dental caries, a chronic disease characterized by tooth decay primarily caused by bacterial action, occupies the second position in terms of disease burden [1]. In the Global Burden of Disease Study 2016, dental caries ranked highest in prevalence and second in incidence globally, following only upper respiratory infections [2]. Various factors contribute to the onset of caries, and microbial factors play a significant role. Among the microbial agents implicated in caries, *S. mutans* is notable because of its acidogenic, aciduric, and biofilm-forming capabilities. The cariogenic effects of *S. mutans* primarily occur through biofilm formation, in which organic acid metabolism within the biofilm leads to tooth demineralization. Glucosyltransferases (Gtfs) play a pivotal role in biofilm formation by synthesizing glucans through sucrose-dependent pathways, thereby providing a matrix for biofilm formation. VicRK signal transduction system regulates Gtfs expression, thereby promoting biofilm formation [3]. In addition, sortase A (SrtA) and antigen I/II (AgI/II) facilitate bacterial adhesion and colonization through sucrose-independent mechanisms, thereby influencing the adherence of virulence-related surface proteins to biofilms [4]. Furthermore, the cariogenic virulence of *S. mutans* is modulated by host oxidative stress responses [5]. Superoxide dismutases (SODs) and VicRK serve as adaptive systems in *S. mutans* and attenuate oxidative stress by preventing the accumulation of reactive oxygen species (ROS), thereby protecting *S. mutans* from oxidative damage [6]. This adaptive response indirectly contributes to the virulence of *S. mutans* against dental caries. All these enzymes introduced above can be considered as specific targets involved in the cariogenic process of *S. mutans*.

Classic strategies for dental caries prevention include fluoride which aids in remineralization, limiting sugar intake and frequency to reduce the substrate for microbial extracellular polysaccharides (EPSs) and acid production, and mechanical removal of dental plaque (brushing and flossing) [[7], [8], [9]]. Fluoride remains the primary method for caries prevention because of its significant reduction in caries incidence. Despite its remineralization effect, fluoride has antimicrobial action against *S. mutans*, which plays an important role in its anti-caries ability. The inhibitory effect of fluoride on intracellular metabolism depends on the influx of hydrogen fluoride (HF), which dissociates to the proton (H) and fluoride ion (F^−^) in the cytoplasm and has a strong pH-dependence. The intracellular F^−^ and H can affect *S. mutans* enzymatic activities and physiological processes like F-adenosine triphosphatase (ATPase) and glycolysis in direct or indirect ways. In these ways, fluoride can affect acid production and acid tolerance and lower the adherence to tooth surfaces of *S. mutans* [10]. Tooth surfaces treated with fluoride can inhibit bacterial acid production at the bacteria/tooth interface by inhibiting the metabolic activity of *S. mutans* cells [11]. Although it can affect streptococcal glycolysis [12], its preventive efficacy against caries is incomplete, and *S. mutans* can develop resistance to fluoride [13]. As an adverse reaction, improper fluoride use can also pose risks, such as fluorosis and fluoride toxicity. Consequently, the development of novel anti-caries holds substantial promise. Our research team is dedicated to discovering such agents, with preliminary experiments developing substances, such as caffeic acid phenethyl ester (CAPE) [14], and the antimicrobial peptide GH12 [15,16]. CAPE and GH12 exhibit bacteriostatic effects against major cariogenic bacteria, suppressing their acid production and showing potential for both anti-caries efficacy and clinical application. However, the lack of targetability against the virulence potential of *S. mutans* and its biofilm state remains a challenge for enhancing its caries control efficacy. Therefore, the development of new selective inhibitors against the virulence targets of the main cariogenic strain *S. mutans*, such as Gtfs and SrtA, may be an important direction for the development of anti-caries treatment.

Drug discovery is a multistage and complex process influenced by various factors that determine its success [17]. On average, drug development takes 12 years and costs up to $2.6 billion [18]. Despite substantial investment, the success rate of clinical trials is only 13% [19], with high attrition rates in drug development. With advancements in computer technology, *in silico* approaches containing computer-aided drug design (CADD) and AI-assisted drug design (AIDD) have been increasingly employed in drug design [20,21]. Computational methods, such as quantitative structure-activity relationships (QSAR), pharmacophore modeling, molecular docking, and molecular dynamics simulations (MDs), provide new tools for designing anti-virulence drugs, markedly increasing their efficiency compared with traditional empirical approaches [22].

**Fig. 1:**
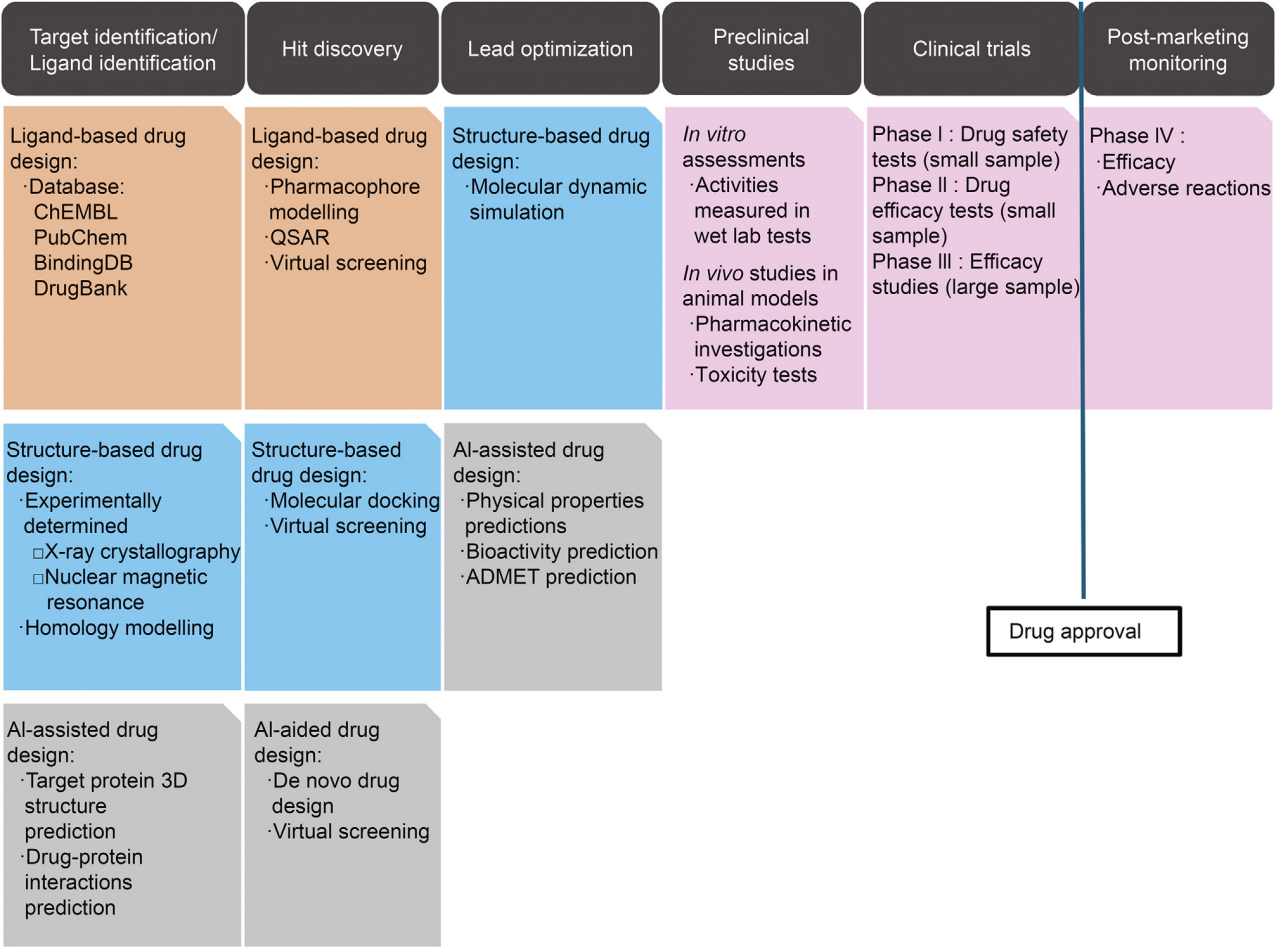
Application of *in silico* approaches in the drug design process. In the target identification and ligand identification phases, computer-aided drug design (CADD) methods like database screening and homology modeling are utilized to acquire needed structure in ligand-based drug design (LBDD) or structure-based drug design (SBDD). For hit discovery, potential compounds are found through methods including pharmacophore modeling, quantitative structure-activity relationships (QSAR), molecular docking, and artificial intelligence (AI)-driven de novo design and virtual screening (VS). Lead optimization focuses on structure-based dynamics simulations and AI methods to predict physical properties, bioactivity, and absorption, distribution, metabolism, excretion, and toxicity (ADMET). Then, preclinical studies in wet lab are assessed to ensure drug-like properties and safety for clinical trial approval. Integrating computer-aided and AI-based methodologies can significantly enhance efficiency. 3D: three-dimension.

The drug development process encompasses target and ligand identification and validation, hit discovery, lead optimization, preclinical studies, approval, and post-marketing monitoring [23]. The *in silico* drug design approaches are used throughout these stages to accelerate drug development and improve efficiency (Fig. 1), and they have been extensively applied in the development of various medications, including antibiotics [24], psychotropic and neurological drugs [25], and anticancer agents [26]. Considerable recent efforts have been undertaken to develop novel targeted anti-virulence agents for the new generation. Many of these attempts have relied on *in silico* approaches, which have become more and more powerful and complementary to biochemical attempts. However, there is a lack of a review focusing on this topic and discussing the developmental trajectory of these methodologies.

The current study aims to summarize the important developments of targeted anti-virulence drug design and to aid future advancements and the practical application of CADD and AIDD in this field. To begin with, we introduce the specific methods and models within CADD and AIDD in drug design. Following this, we delve into the recent progress in the drug design focusing on the targeting of singular or multiple virulence factors of *S. mutans*, including Gtfs, the VicRK system, SrtA enzyme, AgI/II, and SODs. In the last part, we provide an overview of the emerging opportunities as well as the challenges that are currently faced in this domain.

## 2 Strategies in CADD

With the development and application of mathematical modeling and computational drug design methods, CADD has gradually demonstrated advantages in fields such as drug target screening owing to its rapid computational predictive capabilities [27] and relatively cost-effective nature [19]. CADD is a computational method used to discover, develop, and analyze drugs and active molecules with similar biochemical properties [28], making it a critical approach in modern preclinical drug discovery.

**Fig. 2:**
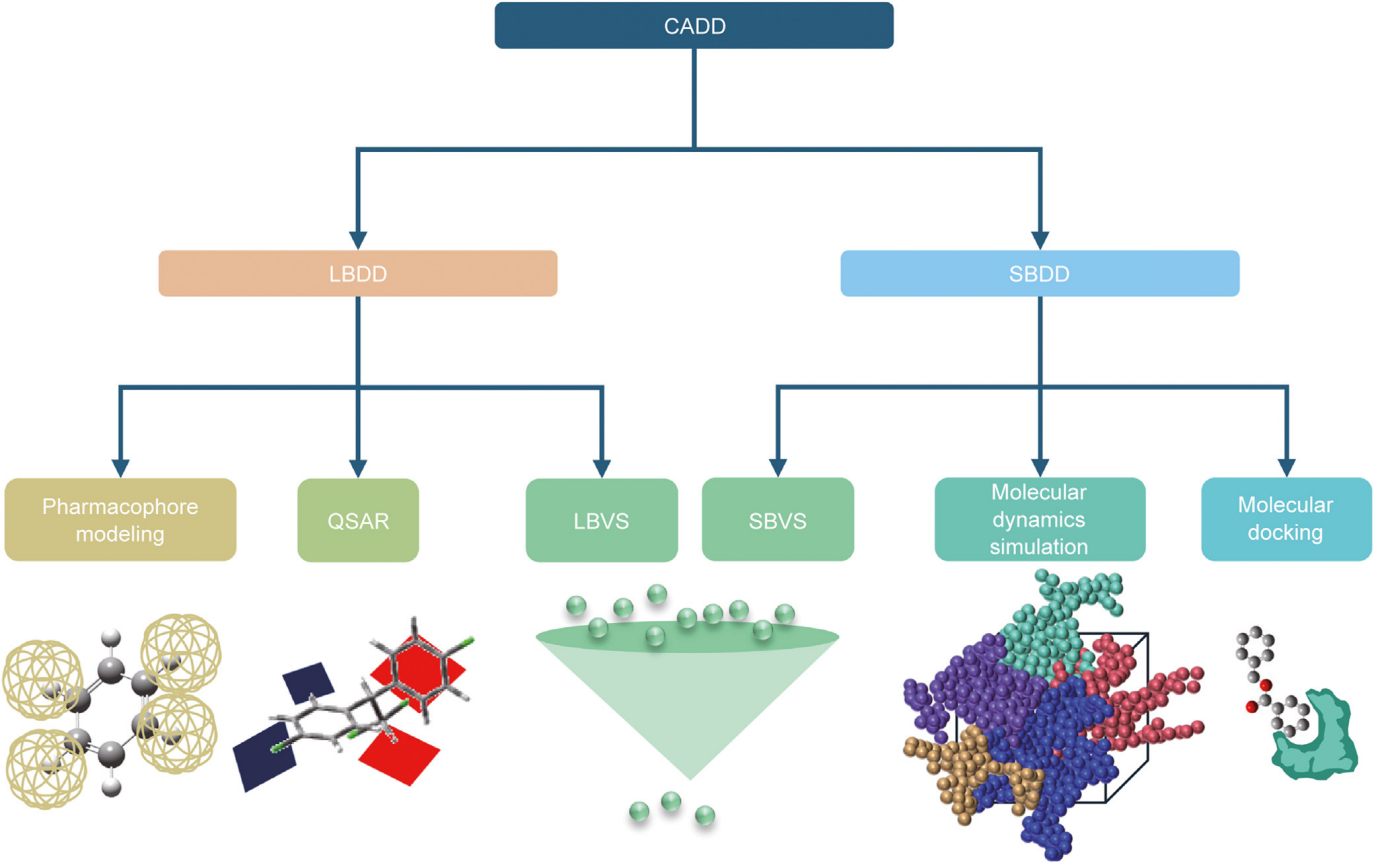
Overview of computer-aided drug design (CADD) methods. CADD can be categorized as ligand-based drug design (LBDD) or structure-based drug design (SBDD) based on the availability of the target protein and ligand structures. LBDD methods include pharmacophore modeling, quantitative structure-activity relationships (QSAR), and ligand-based virtual screening (LBVS). SBDD methods include structure-based virtual screening (SBVS), molecular dynamics simulations (MDs), and molecular docking.

CADD primarily encompasses ligand-based drug design (LBDD) and structure-based drug design (SBDD) [29] (Fig. 2). LBDD methods include pharmacophore modeling and QSAR, which use the relationship between biological activity and chemical structure to identify appropriate lead compound molecules. In contrast, SBDD uses structural information from established protein-binding sites to select molecules for further investigation [25]. CADD uses structural knowledge of targets (SBDD) or known biologically active ligands (LBDD) to identify promising drug candidates [30]. When screening large compound libraries, SBDD and LBDD are often combined to produce more accurate results while reducing computational burden. The complementary use of SBDD and LBDD highlights their strengths and integration with experimental norms, thereby enhancing rational drug design [30].

### 2.1 Ligand-based approaches

LBDD uses known ligands or a set of ligand data to predict the activities of other small molecules based on their common characteristics [25]. This principle is based on the concept of molecular similarity, whereby compounds with similar chemical structures exhibit similar binding properties. LBDD extracts biological and chemical information from known active ligands to identify key features related to the biological activity. This approach can be employed without resolving the target crystal structure or when the drug target is unknown, making it a crucial computational strategy for facilitating and guiding drug discovery in the absence of target molecular structure data [26]. During the phases of target identification and hit discovery, ligand-based approaches such as virtual screening (VS) can expedite the evaluation of potential hit compounds. In the subsequent lead optimization stage, three-dimensional (3D) QSAR and pharmacophore modeling techniques within LBDD can be employed to identify more suitable molecular configurations (Fig. 1).

#### 2.1.1 Pharmacophore modeling

A pharmacophore is an abstract description of the structural properties essential for a biomolecule to recognize a ligand [31]. Pharmacophore models represent a collection of chemical features that define the interactions of ligands with biological targets and elucidate physiological responses [25]. Through pharmacophore-based methods, the molecular functional features required for binding to a specific receptor are defined, thereby guiding extensive VS of compounds to select optimal candidates [27].

#### 2.1.2 QSAR

QSAR is a computational and statistical tool used to interpret the observed structural changes resulting from substitutions [31], making it a significant method in drug design. QSAR relies on various molecular descriptors or fingerprints (FPs) to analyze the biological activity of drugs [26]. QSAR predicts activity by statistically correlating molecular descriptors with biological data such as binding affinity (dissociation constant, *K*_*D*_) or potency (half-maximal effective concentration (EC_50_) or half-maximal inhibitory concentration (IC_50_) values) [25]. Unlike pharmacophore models, QSAR models quantitatively measure the biological activity and can identify positive or negative effects based on specific molecular features [26]. With advancements in QSAR methodologies, the field has evolved from two-dimensional (2D) QSAR to 3D QSAR and multi-dimensional QSAR [32], transitioning from single-model approaches to combinatorial and hybrid QSAR frameworks [33]. These methodologies have found applications across diverse fields, including medicinal chemistry, materials science, and predictive toxicology. However, QSAR modeling has limitations; insufficient molecules in the training set may limit its ability to accurately predict all properties, thus affecting its efficacy in identifying the most effective compounds.

#### 2.1.3 Ligand-based virtual screening (LBVS)

VS is a computational method used in simulations to select promising compounds from chemical databases and resembles a computerized experimental biology evaluation approach [31]. There are two primary types of VS: structure-based virtual screening (SBVS) (also known as receptor- or target-based VS, discussed in the section “Structure-based approaches” below) and LBVS. LBVS is conducted when the 3D structure of a protein target is unavailable instead of relying on the structural activity data of known active molecules [28]. Its main limitation lies in its sensitivity to deviations from the reference templates, which may lead to an overfitting of the input structures.

### 2.2 Structure-based approaches

SBDD involves the direct design of drugs with specific targets, relying on known structural information to determine the effects of the interactions between bioactive compounds and their respective receptors [26]. Techniques such as X-ray and nuclear magnetic resonance spectroscopy (NMR), as well as resources such as the Protein Data Bank (PDB), provide the necessary protein 3D structures for SBDD [34]. When the target protein structure is unknown, SBDD can use methods such as homology modeling, threading, and de novo calculations to predict and construct the required protein structures [31]. Molecular docking, VS, and MDs are the fundamental methods used in SBDD. VS based on structure methods can also be employed during the target identification and hit discovery phases. Beyond these initial steps, molecular docking and MDs within SBDD offer significant advantages for the optimization of lead compound configurations (Fig. 1).

#### 2.2.1 Molecular docking

Molecular docking is a typical structure-based approach in rational drug design and is widely recognized as the most extensively applied method in CADD [31]. Molecular docking predicts and studies the binding modes and affinity interactions between ligands and receptor biomolecules [26]. It can explore ligand conformations within the binding site of macromolecular targets by estimating the ligand-receptor binding free energy and assessing the critical phenomena involved in molecular recognition processes through programs like AutoDock [35]. Molecular docking emphasizes rapid and efficient assessment, typically employing a “rigid docking” approach that considers static geometric, physical, and chemical complementarity between ligands and target proteins while disregarding flexibility and induced fit theory.

#### 2.2.2 MDs

Molecular docking alone often fails to provide precise insights into the conformation of drug-target complexes. Therefore, MDs of complex systems are required. MDs are based on Newton's equations of motion, guiding interactions, and yielding stable receptor-ligand conformations from molecular dynamic trajectories [31]. Because MDs inherently model protein dynamics, they can effectively elucidate structural changes within proteins [25]. Using MDs, one can evaluate complex stability, atomic-level interactions, system fluctuations, and binding free energies, which serve to refine and validate the docking outcomes of protein-ligand complexes [28].

#### 2.2.3 SBVS

SBVS relies on the availability of 3D data concerning biological targets to predict the optimal interaction patterns between two molecules to form stable complexes. It uses scoring functions to estimate noncovalent interactions between ligands and molecular targets [36]. VS strategies often integrate both ligand-based and structure-based techniques by employing sequential, parallel, or hybrid methods to enhance the success rate of hit identification [37]. In VS applications, experimental validation is paramount, and assays can be used to test hypotheses. The calibration of theoretical results against experimental outcomes is crucial and serves as a benchmark for evaluating and cross-verifying computational models [28].

SBDD and LBDD are applied to each step of CADD. The main differences between SBDD and LBDD in drug discovery applications are the availability of receptor or ligand structures, and whether drug design is based on the principle of binding to receptor and ligand or on the principle of ligand structure. Both approaches enable the design of featured potential drug lead compounds.

## 3 Classical methods in AIDD

In drug development, artificial intelligence (AI) plays a crucial role in identifying hits and lead compounds, expediting drug target validation, and optimizing drug structural design like CADD. AI can also assist in predicting the 3D structures of target proteins, protein-protein interactions, drug activity, and de novo drug design [38]. Over the past decade, the volume of available biomedical data has grown at an unprecedented rate [24], propelling drug design and discovery into an era of big data. The advancement of automation technologies and the exponential growth of data have encouraged the use of machine learning (ML) and deep learning (DL) techniques to mine this vast amount of data and extract valuable patterns [39]. This further promotes the integration of AI with computer-aided drug discovery technologies, thereby accelerating the design and discovery of novel drugs [40].

### 3.1 ML

ML is a branch of AI and computer science that focuses on using data and algorithms to simulate human learning processes, thereby progressively improving accuracy. ML is categorized into four types based on the learning methods: supervised learning, unsupervised learning, semi-supervised learning, and reinforcement learning [31]. They employ statistical methods and trained algorithms to generate classifications or predictions, thereby providing key insights into data mining. Common ML algorithms utilized in drug discovery include support vector machines (SVM), random forests (RF), and k-nearest neighbors (KNN), among others. Notably, SVM is distinguished by its effectiveness in handling high-dimensional variables in small sample sizes, making it suitable for diverse modeling applications within the drug discovery process [41]. RF is employed for various classification tasks and target identification by constructing multiple decision trees for ensemble operations [42]. Meanwhile, KNN performs classification and regression by determining the class of the k nearest neighbors in the feature space, often demonstrating superior performance when combined with other algorithms compared to its standalone application [43]. The key tasks of AI algorithms on extensive datasets include classification, regression, clustering, and pattern recognition [44]. ML has been extensively applied across all phases of preclinical drug discovery (Fig. 1), facilitated by advancements in algorithmic techniques and the ongoing integration of time-efficient methodologies in pharmaceutical research. During the drug screening phase, various ML algorithms, such as RF and SVM, can be utilized to evaluate compound libraries and identify potential candidates. Additionally, ML algorithms can be employed to calculate molecular descriptors for drug compounds, which are instrumental in predicting various physical and chemical properties and guiding the design of drug structures [45].

Ruthenium complexes have advantageous properties such as high antimicrobial activity, low cytotoxicity, and low hemolytic rates. Orsi et al. [46] employed classical ML models, including RF, naïve bayes (NB), SVM, multilayer perceptron (MLP), extreme gradient boosting (XGBoost), and KNN algorithms, to train models on a library containing 288 ruthenium arene Schiff base complexes, which performed well and were effectively used to screen for novel ruthenium complexes against methicillin-resistant *Staphylococcus aureus* (MRSA). Huang et al. [47] developed a pipeline that combines different ML algorithms to integrate empirical judgment, classification, ranking, and regression tasks. This pipeline was used to identify potential antimicrobial hexapeptides and also demonstrated applicability in screening heptapeptides, octapeptides, and nonapeptides, showing robust generalization capabilities.

### 3.2 DL

DL is a branch of ML that employs complex architectures with multiple layers and nonlinear transformations of abstract data. DL surpasses traditional ML methods by using several layers of processing units, called neurons, to make predictions based on extensive multidimensional datasets. In the field of drug design and discovery, prominent applications include convolutional neural networks (CNNs), recurrent neural networks (RNNs), long short-term memory (LSTM) networks, and multitask learning (MTL) [31]. Compared with basic ML algorithms, DL excels in iterative self-learning. It is not only suitable for compound classification and activity prediction but also for generating models such as generative adversarial networks (GANs), variational autoencoders (VAEs) and wasserstein autoencoder (WAE) [48] to autonomously generate molecules for de novo compound design. DL is well-suited for processing large datasets and has found applications across various domains of drug discovery, including compound screening, descriptor calculation, and the prediction and analysis of physical and chemical properties, as well as model optimization and guidance [49]. Furthermore, DL has shown promise in compound image recognition and unknown structure generation [50]. Theoretically, with adequate training datasets and sufficient computational resources, DL can outperform traditional ML; however, it is constrained by the quality of available data [51]. Additionally, the “black box” nature of current DL algorithms hampers the understanding of their underlying mechanisms, leading to high predictive performance but limited interpretability regarding molecular processes, which impedes broader applications in medicine. To address these challenges, researchers have undertaken numerous studies aimed at elucidating DL models, such as employing ensemble approaches instead of single models [52] and integrating biological knowledge into the modeling process [53], both of which have yielded promising results.

Das et al. [54] constructed a deep-learning-based generative model using the VAE/WAE framework, in which the peptide generation is formulated as a density modeling problem, and applied an LSTM classifier model, a RNN having high performance in solving the vanishing gradient problem, for activity prediction. Within 48 day, they identified two compounds that demonstrated antibacterial efficacy against various gram-positive and gram-negative pathogens. These compounds also exhibit a low propensity for inducing resistance and low toxicity, highlighting their potential for accelerated discovery of antimicrobials by integrating DL with MDs.

AIDD can perform all functions of CADD throughout the drug development process, often achieving superior results with sufficient high-quality training data. Additionally, AIDD offers significant advantages in generating compound molecular structures, elucidating drug synthesis pathways, and predicting pharmacokinetic performance [31]. However, AI methods have certain limitations. The effectiveness of the algorithms relies heavily on the availability of comprehensive, high-quality datasets, which directly affects their performance. Several advanced models that leverage machine-learning capabilities often lack transparency because of their “black-box” nature, making it challenging to interpret and apply their results in a rational manner. Consequently, these drawbacks restrict their potential application in rational drug discovery and design [55,56].

## 4 Application in targeted anti-virulence drug design

Currently, the most widely used caries control agent in clinical practice is still fluoride [57]. However, these traditional anti-virulence agents have several drawbacks. For instance, an overdose of fluoride can lead to toxicity [57]. More importantly, these drugs are broad-spectrum antimicrobials that do not specifically target the main cariogenic bacteria or specific targets involved in the caries process. Considerable progress has been achieved in developing new generations of novel targeted anti-virulence agents that heavily rely on *in silico* approaches. The *in silico* approaches, especially CADD techniques such as molecular docking and VS, are widely applied in this domain, and the validated druggable targets primarily are biofilm-related enzymes, such as Gtfs, AgI/II, SrtA, VicRK system, and oxidative stress-related SODs.

### 4.1 Gtfs-targeted inhibitors

**Fig. 3:**
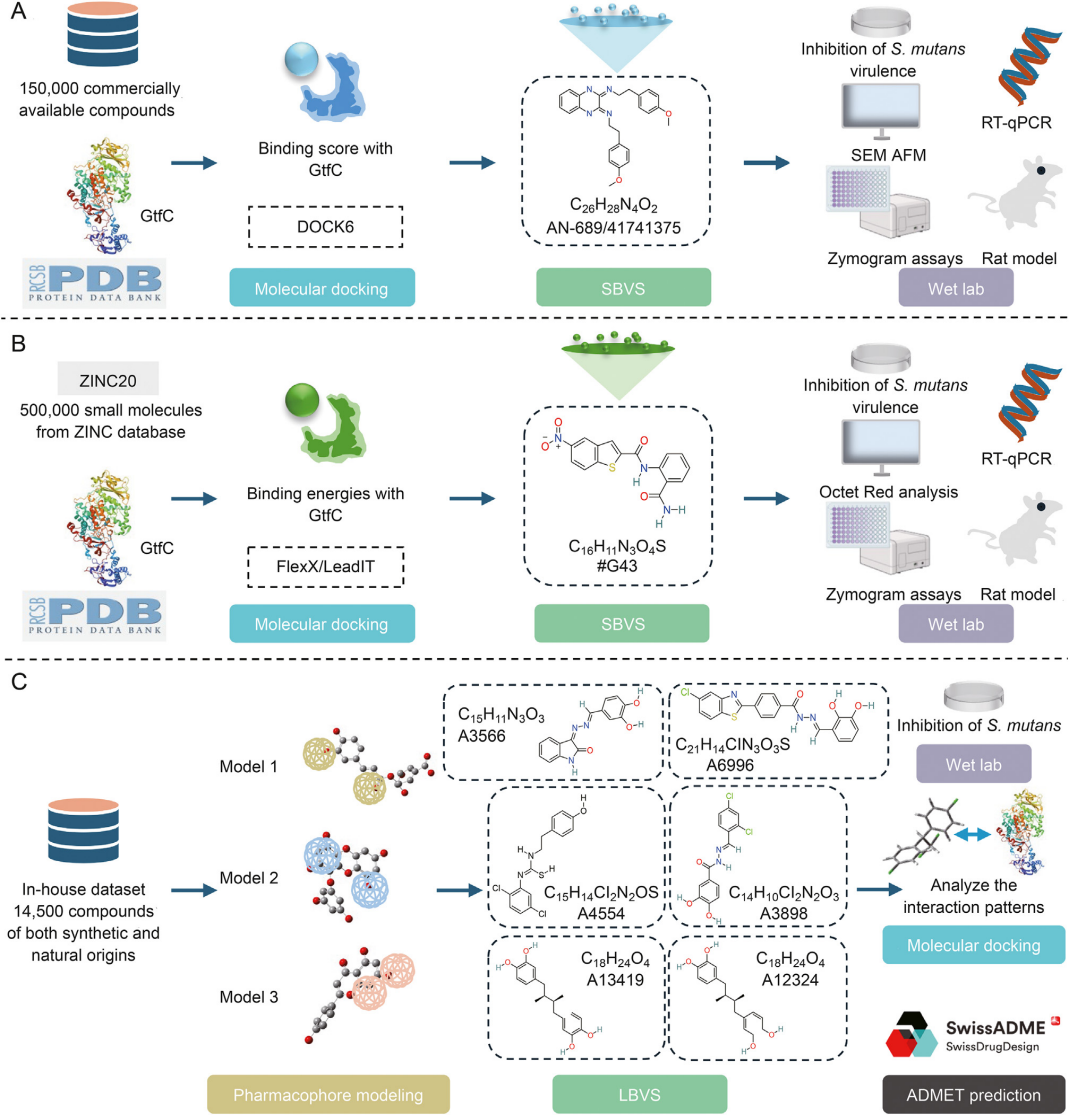
Strategies for applications of *in silico* approaches targeting glucosyltransferases (Gtfs). (A) 2-(4-methoxyphenyl)-N-(3-{[2-(4-methoxyphenyl)ethyl]amino}-1,4-dihydro-2-quinoxalinylidene)ethanamine was identified through structure-based virtual screening (SBVS) and molecular docking against GtfC. (B) Compound #G43 was determined via SBVS and molecular docking against the catalytic domain of GtfC complexed with acarbose. (C) Six potential anti-caries compounds (A3566, A6996, A4554, A3898, A13419, and A12324) were discovered through pharmacophore modeling and ligand-based virtual screening (LBVS). Then the interaction with GtfC was analyzed by molecular docking, and the artificial intelligence (AI)-based absorption, distribution, metabolism, excretion, and toxicity (ADMET) prediction tool SwissADME was used to predict its pharmacokinetics and toxicity. S*treptococcus mutans*: *S. mutans*; SEM: scanning electron microscopy; AFM: atomic force microscopy; RT-qPCR: reverse transcription quantitative polymerase chain reaction.

Gtfs are exoenzymes that play a significant role in the pathogenesis of *S. mutans*-mediated dental caries, including early childhood caries. *S. mutans* expresses at least three genetically distinct Gtfs: GtfB primarily synthesizes insoluble glucans rich in α-1,3 linkages, GtfD synthesizes soluble glucans with α-1,6 linkages, and GtfC produces a mixture of insoluble and soluble glucans containing α-1,6 linkages [58]. These membrane-associated and surface-exposed Gtfs, including GtfB, GtfC, and GtfD, synthesize glucans that form EPSs that are critical for biofilm formation. EPSs provide a water-insoluble matrix and create an acidic environment that facilitates colonization by cariogenic bacteria. In addition, the EPS-rich extracellular matrix protects microorganisms from antimicrobial treatment and confers viscoelasticity to biofilms, thereby hindering their mechanical removal [13]. Targeted inhibitors of Gtfs can selectively reduce the virulence of *S. mutans* without affecting their presence in the oral cavity or other species. Therefore, Gtfs are attractive targets for effective therapeutic interventions aimed at inhibiting cariogenic biofilm formation. Currently, several drugs targeting Gtfs have been designed using various strategies (Fig. 3).

Ren et al. [59] targeted GtfC from *S. mutans* as a receptor for molecular docking studies using its crystal structure. They employed the DOCK6 software to conduct SBVS of the ZINC database and screened approximately 150,000 commercially available compounds. They identified the quinoxaline derivative 2-(4-methoxyphenyl)-N-(3-{[2-(4-methoxyphenyl)ethyl]imino}-1,4-dihydro-2- quinoxalinylidene)ethanamine as a potent Gtfs inhibitor. *In vitro* experiments confirmed that this quinoxaline derivative effectively inhibited the synthesis of insoluble glucans (60%) and biofilm formation (79%). Enzymatic assays demonstrated that the compound antagonized both soluble-phase GtfC and GtfD. In the rat caries model, the compound significantly reduced the incidence and severity of smooth surfaces and fissure caries while also decreasing the percentage of *S. mutans* in the dental plaque. These findings highlight that this compound is a promising Gtfs-targeted inhibitor capable of inhibiting biofilm formation and cariogenicity [59].

Using the crystal structure of the GtfC catalytic domain complexed with acarbose, Zhang et al. [22] conducted SBVS of 500,000 drug-like compounds using the FlexX/LeadIT software. Based on predicted binding affinities and drug-like properties, 90 compounds with diverse scaffolding structures were obtained. These compounds were evaluated through *in vitro* biofilm assays using cariogenic *S. mutans* to assess their ability to reduce *S. mutans* biofilm formation and inhibit Gtfs activity. They identified seven effective biofilm inhibitors, among which the lead compound #G43 selectively inhibited the formation of *S. mutans* biofilms without impacting the overall growth of cariogenic and commensal oral bacteria. #G43 has emerged as a promising candidate for caries prevention, highlighting the efficacy and potential of designing independent inhibitors targeting GtfC against cariogenic *S. mutans* [22].

Different Gtfs inhibitors exhibit varying chemical scaffold characteristics and may target different sites and mechanisms of action. Atta et al. [60] used ligand-based pharmacophore modeling methods to identify potential targets for the inhibition of biofilm formation using different Gtfs inhibitors. Three pharmacophore models based on the combinations of 5-o- caffeoyl shikimic acid and *p*-coumaric acid were established (Model 1), eckol and epicatechin (Model 2), and apigenin and caffeic acid (Model 3). Each model shows different efficacy characteristics due to the different ligand structures used to construct the models. These models were then used to screen internal compound databases containing 14,500 synthetic and natural compounds. Molecular docking was employed to analyze intermolecular interactions, resulting in the identification of six compounds exhibiting strong binding affinities. The pharmacokinetic profiles, including adsorption, distribution, metabolism, excretion, toxicity (ADMET) properties of the compounds, were evaluated using the SwissADME and Protox-II servers, followed by *in vitro* biofilm formation assays. These compounds demonstrated potent antibiofilm activity and favorable pharmacokinetic characteristics, suggesting their potential as foundational derivatives in the development of novel and effective treatments for dental caries [60].

### 4.2 AgI/II-targeting inhibitors

**Fig. 4:**
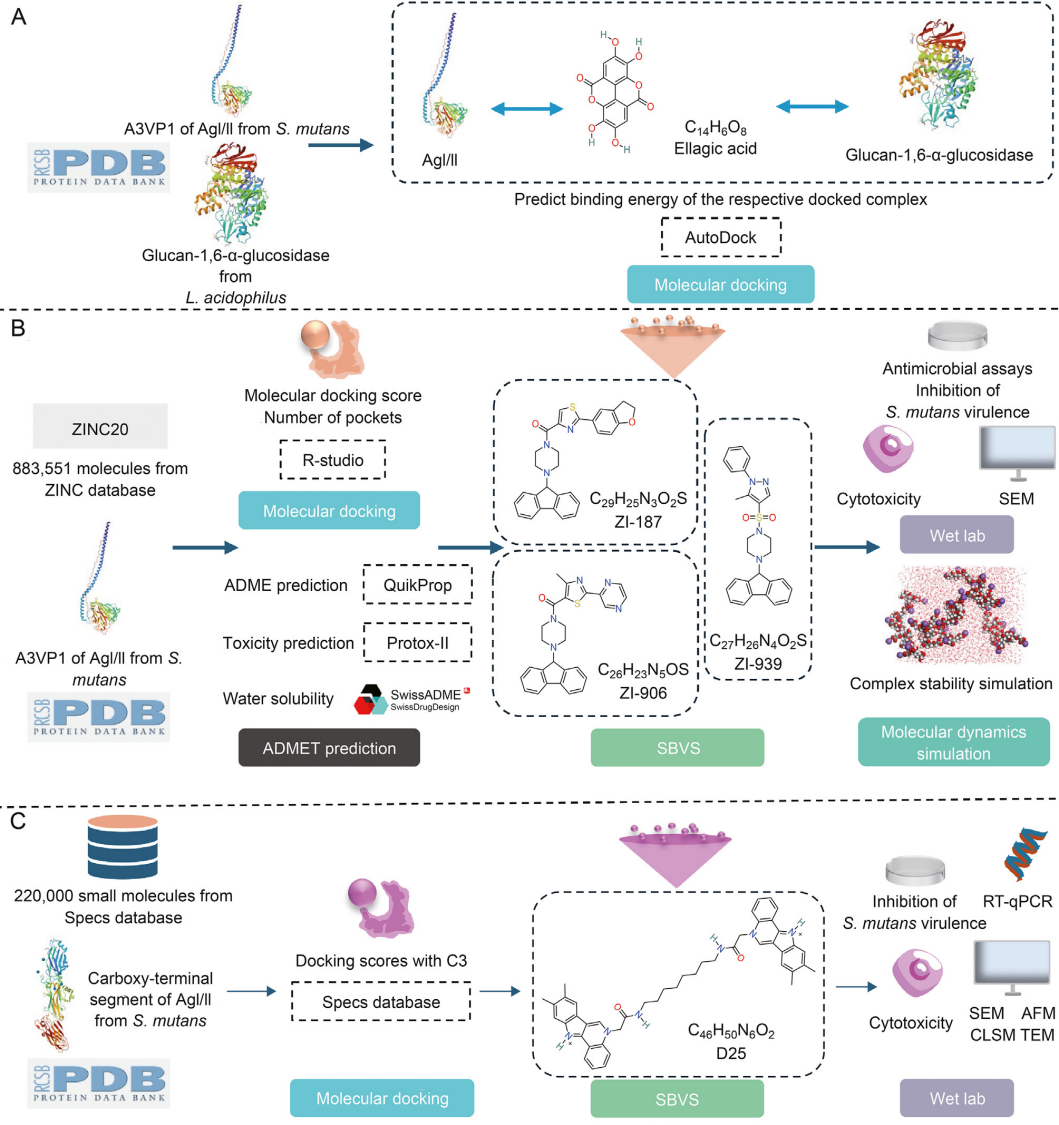
Strategies for applications of *in silico* approaches targeting antigen I/II (AgI/II). (A) Ellagic acid was identified through molecular docking. (B) Molecular docking structure-based virtual screening (SBVS) and molecular dynamics simulations (MDs) were employed to assess ZINC19835187 (ZI-187), ZINC19924906 (ZI-906), and ZINC19924939 (ZI-939). (C) Compound D25 exhibited selective inhibition against cariogenic biofilms through molecular docking and SBVS, establishing it as a potential lead compound for anti-caries applications. *Streptococcus mutans*: *S. mutans*; *Lactobacillus acidophilus*: *L. acidophilus*; ADMET: absorption, distribution, metabolism, excretion, and toxicity; SEM: scanning electron microscopy; RT-qPCR: reverse transcription quantitative polymerase chain reaction; AFM: atomic force microscopy; CLSM: confocal laser scanning microscopy; TEM: transmission electron microscopy.

AgI/II, also known as SpaP, Pac, or P1, exists in most *Streptococcus oralis* strains and is an important adhesin involved in biofilm formation. It participates in adhesion and bacterial co-aggregation during early biofilm formation [61]. In the absence of sucrose, *S. mutans* produces several important adhesins, such as AgI/II. These adhesins combine with a glycoprotein, salivary agglutinin (SAG), to form sucrose-independent cell adhesions and participate in biofilm formation [62]. At present, several pharmaceutical agents targeting AgI/II have been developed employing diverse strategies (Fig. 4).

Chittrarasu et al. [63] applied a high-throughput VS approach based on this structure. The optimized ligand ellagic acid was docked with the crystal structure of AgI/II A3VP1 of *S. mutans* and the structure of glucan-1,6-α-glucosidase from *Lactobacillus acidophilus* respectively using AutoDock. The binding energies of the ellagic acid-*S. mutans* docking complex and ellagic acid-*L. acidophilus* docking complex were calculated as −10.63 and −7.30 kcal/mol, respectively, identifying ellagic acid as a potential inhibitor in the treatment of dental caries through the targeting of both *S. mutans* and *L. acidophilus* [63].

Using a SBVS method, Rivera-Quiroga et al. [64] screened over 883,551 molecules from the ZINC database and performed cytotoxicity analysis on fibroblasts, *S. mutans* adhesion studies, scanning electron microscopy (SEM) analysis of bacterial integrity, and MDs. They identified three molecules, ZINC19835187 (ZI-187), ZINC19924906 (ZI-906), and ZINC19924939 (ZI-939), which inhibited the adhesion of *S. mutans* to polystyrene microplates by approximately 90% without exhibiting cytotoxic activity. Specifically, ZI-187 showed a stable interaction with AgI/II (PDB: 3IPK), suggesting that it is a novel molecule that targets AgI/II and serves as a potential antimicrobial agent against *S. mutans* [64].

The C123 segment of the AgI/II (P1 protein) forms amyloid fibrils within *S. mutans* biofilms. The C3 segment plays an important role in the C123-P1 interaction and is expected to be an anti-amyloid target [65]. Chen et al. [66] used the crystal structure of C123 from the PDB protein database, using the available C3 segment as the target protein for SBVS using the molecular operating environment (MOE) software. The binding energies of the C3 segment to small molecules were obtained from the Specs database. After calculating the data within the predicted binding pocket, prospective small molecules with drug-like properties were identified using Lipinski's rule. The top 99 small molecules ranked by their binding affinity were selected, including 55 dimethyl sulfoxide (DMSO) soluble compounds, named D1–D55, for crystal violet staining. Among them, D25 selectively inhibited biofilm formation by *S. mutans* but showed no significant impact on biofilm formation by *Streptococcus gordonii* and *Streptococcus sanguinis*. The expression of amyloid-related genes is upregulated upon stimulation with D25. In conclusion, D25 is a promising antibacterial agent with the ability to affect amyloid fibers and inhibit *S. mutans* biofilm formation [66].

### 4.3 SrtA-targeting inhibitors

**Fig. 5:**
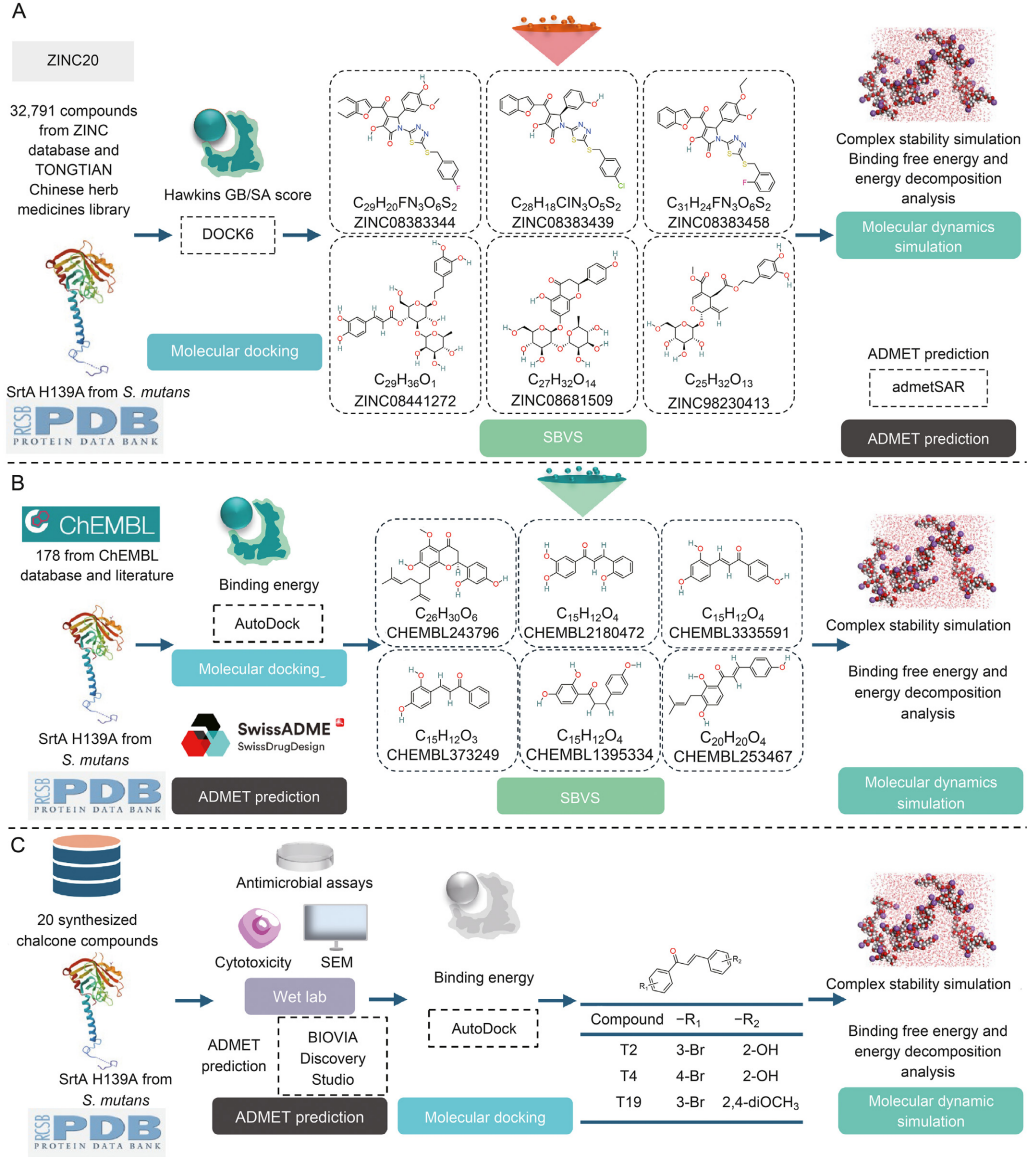
Strategies for applications of *in silico* approaches targeting sortase A (SrtA). (A) Compounds such as ZINC08383344, ZINC08383439, ZINC08383458, ZINC08441272, ZINC08681509, and ZINC98230413, composed of benzofuran, thiadiazole, and pyrrole scaffolds, have demonstrated favorable affinity for SrtA and pharmacokinetic properties through structure-based virtual screening (SBVS) and molecular dynamics simulations (MDs). (B) Compounds identified through molecular docking and SBVS methods, including CHEMBL243796, CHEMBL2180472, CHEMBL3335591, CHEMBL373249, CHEMBL1395334, and CHEMBL253467, exhibit superior anti-caries efficacy compared to chlorhexidine through MDs methods. (C) T2, T4, and T19, identified as chalcone scaffold compounds through structure-based drug design (SBDD) methods like molecular docking, show high sensitivity towards SrtA by MDs and hold promise for development as novel anti-caries agents. ADMET: absorption, distribution, metabolism, excretion, and toxicity; SEM: scanning electron microscopy.

Sortases are cysteine transpeptidases that mediate the covalent attachment of proteins to the microbial cell wall and are crucial for microbial structure. Widely present in gram-positive bacteria, sortases anchor surface proteins containing LPXTG and NPQTN motifs to the cell wall, making them promising targets for antimicrobial drug development [67]. Sortases are encoded by all gram-positive bacteria, a few gram-negative bacteria and archaea, and are classified into types A, B, C, D (D1 and D2), E, F6, and “marine sortases” [68]. SrtA is a membrane-associated cysteine protease that catalyzes cell wall sorting reactions and is responsible for covalently attaching virulence-associated surface proteins to host tissues. SrtA anchors virulence-associated adhesins to host tissues, plays a critical role in biofilm formation during caries development, and is a potential target for the development of antimicrobials and anti-virulence agents to prevent biofilm-related infections [4]. Current strategies used to identify novel potential SrtA inhibitors include natural product screening, small-compound library high-throughput screening, computer VS, and fragment-based lead compound discovery [4]. Currently, various studies are employing CADD methods for the discovery of drugs targeting SrtA (Fig. 5).

Luo et al. [69] used a high-throughput VS method to identify new potential SrtA inhibitors in *S. mutans*. They used DOCK6 and AutoDock for molecular docking and screened potential SrtA inhibitors from the Specs library of the ZINC database and the TONGTIAN library. Nine compounds were selected and subjected to further MDs using the Gromacs software combined with free energy analysis and evaluation of ADMET properties. The results showed that several similar compounds composed of benzofuran, thiadiazole, and pyrrole had good affinities and suitable pharmacokinetic parameters, suggesting their potential as inhibitors of SrtA catalysis. This could provide a new strategy for treating microbial infectious diseases [69].

Salmanli et al. [70] used a structure-based method to screen 178 compounds in the ChEMBL database to study their inhibitory effects of different compounds on *S. mutans*-SrtA-mediated biofilm formation. AutoDock was used for molecular docking analysis of SrtA enzymes and compounds. The results showed that six compounds (78, 10, 75, 12, 17, and 97) exhibited better affinity and more favorable pharmacokinetic parameters than the positive control chlorhexidine. Among these, kurarinone (compound 78) was the most active against SrtA, with the lowest binding free energy. This compound forms hydrogen bonds with Arg213 and Cys205 at the binding site of *S. mutans*’ SrtA and plays a crucial role in its inhibitory mechanism. Subsequent MDs suggested that the strong interaction of potential inhibitors with Arg213 disrupted SrtA catalytic activity, thereby reducing the binding of surface proteins to cells. ADME predictions indicated that these effective compounds possessed favorable pharmacokinetic properties compared with chlorhexidine, highlighting their potential for developing novel inhibitors that target biofilm formation [70].

Chalcone is the most abundant secondary metabolite among phenolic compounds in plants and is the precursor of flavonoids in biological pathways; it is primarily found in pesticides, sunscreens, food additives, and pharmaceutical flavoring agents, and has a wide range of biological activities [71]. Previous studies have shown that chalcone scaffold compounds exhibit highly reliable and effective SrtA enzyme inhibition [72]. Yilmaz et al. [73] studied 20 hydroxyl-, nitro-, bromo-, fluoro-, and methoxy-substituted chalcone compounds synthesized *in vitro* by means of molecular docking analyses and MDs, and screened out three precursor compounds, T2, T4, and T19, which showed better activity against *S. mutans* than chlorohexidine. The results of the ADMET evaluation showed favorable pharmacokinetics and great promise as a candidate compound for potential antimicrobial activity, which is expected to lead to the development of SrtA enzyme inhibitors that are more selective than existing inhibitors and have fewer side effects [73].

### 4.4 VicRK-targeting inhibitors

The two-component signal transduction system (TCS) is a crucial regulatory network that enables bacteria to adapt, survive, and express virulence in response to changes in their external environment. The VicRK system is one of the 13 recognized TCS of *S. mutans*. The conservative function of the VicRK signal transduction system is a key regulator of the bacterial oxidative stress response, acidification, cell wall metabolism, and biofilm formation and has been the focus of research in recent years [74]. VicK is a histidine protein kinase containing an atypical PAS domain, which has both self-kinase and phosphatase activities. VicK can undergo autophosphorylation *in vitro* and regulate biofilm formation by influencing the downstream regulatory proteins CovR and VicR [3]. VicR acts as a phosphorylated receptor protein *in vitro* and as a regulatory factor that positively regulates the expression of Gtfs by binding to the promoter regions of GtfB and GtfC, thus promoting biofilm formation [75]. Therefore, the VicRK system is a promising target for Gtfs inhibition.

Silveira et al. [76] identified three binding sites for VicK, S1, S2, and S3, using PockDrug and FTMap analyses. They conducted SBVS using AutoDock 4.2 to screen catechin ligands against the VicK protein. Subsequently, molecular docking was performed at each of the three binding sites, and the docking results were integrated with ligand efficiency indices to assess the binding affinities. The results showed that catecholic compounds, such as MFCD00006830, MFCD00066625, MFCD00017746, MFCD00075939, and MFCD00017413, can potentially inhibit EPS synthesis. These compounds hold promise as novel anti-virulence and antimicrobial agents [76].

In a study on the influence of thyme essential oil on the growth and pathogenic mechanisms of *S. mutans*, Park et al. [77] used molecular docking to analyze the interactions between the potentially active components of thyme and the functional domains and allosteric sites of virulence-related proteins. They found that phenolic components, such as carvacrol and thymol, have an affinity for proteins, such as brpA, vicR, gtfB, gtfC, gtfD, gbpB, and relA, forming stable complexes. This suggests a potential mechanism through which thyme essential oil inhibits the cariogenic functions of *S. mutans*, including acid production, adhesion, and biofilm formation. Research indicates that thyme essential oil can be used in oral healthcare products as a promising anti-caries component [77].

### 4.5 SODs-targeting inhibitors

Bacteria in dental biofilms, such as *S*. *sanguinis*, may produce ROS, such as hydrogen peroxide, causing changes in the microenvironment. Redox reactions are crucial for maintaining ROS metabolic balance. *S. mutans*, the principal cariogenic bacterium in dental caries, is a facultative anaerobe that lacks a complete electron transport chain and thus cannot perform oxidative phosphorylation. Its cariogenic virulence is affected by oxidative stress owing to an incomplete electron transport chain that cannot sustain oxidative phosphorylation. *S. mutans* prevents the accumulation of ROS, mostly through protection systems. As one of the most studied adaptive mechanisms for investigating the role of oxidative stress in *S. mutans*, SODs can directly convert harmful superoxide radicals into H_2_O_2_ and O_2_. Using catalase/peroxidase, the latter breaks down quickly to eliminate superoxide anions, which play important roles in the survival, normal metabolism, and oxidative stress of *S. mutans* [6].

*S. mutans* is the main microaerobic involved in the development of dental plaque and has a single cambial superoxide dismutase (*Sm*SOD) to combat ROS. Cerchia et al. [78] conducted a VS study using the existing 3D structure of *Sm*SOD to identify potential *Sm*SOD inhibitors that interfere with the formation of *Sm*SOD biofilms. Among the screened molecules, ALS-31 exhibited inhibitory effects on *Sm*SOD as well as on typical SOD activities derived from bacterial and eukaryotic sources. The Fe/Mn ratio at the active site of *Sm*SOD influences the inhibitory potency of ALS-31. Moreover, ALS-31 inhibits the activity of other SOD. In the presence of ALS-31, the compound caused the dissociation of the *Sm*SOD homodimer into its monomers, thereby impairing the enzyme's catalytic activity. A docking model was established for ALS-31 at the *Sm*SOD homodimer interface. An initial primer optimizer allowed the identification of a derivative, ALS-31-9, with a 2.5-fold inhibition capacity. ALS-31 can inhibit planktonic growth and biofilm formation of *S. mutans*, whereas a derivative of ALS-31 can inhibit biofilm formation, which opens the way for the design of anticariogenic agents in the future [78].

### 4.6 Multitarget inhibitors

Dental caries is primarily caused by the formation of biofilms dominated by *S. mutans*, involving processes such as EPS production and bacterial aggregation adhesion. Multiple targets, including Gtfs, VicRK system, SrtA, and AgI/II, play crucial roles in biofilm formation. Intervention or inhibition at any stage or target of biofilm maturation can affect the formation and carcinogenicity of dental plaques. Sangavi et al. [79] used high-throughput VS with a fatty acids database called Lipid Metabolites and Pathways Strategy (LIPID MAPS) and performed MDs with the selected protein-ligand complexes. The binding energies were predicted using Molecular mechanics Poisson-Boltzmann surface area (MM/PBSA). In addition, the drug-likeness and pharmacokinetic properties of the ligands were analyzed. In the VS of 46,200 fatty acid molecules (FAs) against three protein targets (GtfC, AgI/II, and SrtA), the top five targets for each protein were screened based on interaction energy. Two common FAs (LMFA01050418 and LMFA01040045) were selected and analyzed for their high binding affinities to AgI/II and SrtA, which could be potential therapeutic drugs targeting the SrtA enzyme [79].

CADD such as VS, molecular docking, and MDs have been applied to the design of targeted anti-virulence agents. This has significantly advanced the development of drugs with better anti-caries potential and the screening and design of antibacterial lead compounds targeting plaque biofilm formation or the oxidative stress process of cariogenic bacteria. However, there are also shortcomings, as most research on targeted antibacterial agents typically focuses only on a certain antibacterial target. For antibacterial drugs, unlike common bacteria such as *S*. *aureus*, there are relatively few studies on cariogenic bacteria. In addition, databases with anti-caries indicators are limited, which may be one reason why AIDD is rarely used in anti-caries drug development.

## 5 Conclusions and future remarks

Traditional drug discovery methods rely on empirical approaches and chemical analyses, which are time-consuming and expensive. Both SBDD and LBDD in CADD significantly reduce the drug discovery cycles and costs. AIDD applies AI algorithms to drug discovery, further reducing manual intervention and enhancing drug discovery [39]. However, both CADD and AIDD require high-quality databases. Currently, available open-access databases suffer from inconsistencies in standards and difficulties in accessing comprehensive information, necessitating further refinement [80].

CADD is currently being applied in the development of inhibitors targeting Gtfs, AgI/II, SrtA, VicRK, and SODs. Using VS combined with molecular docking and MDs, CADD methods have become predominant in this field. Despite its advantages, CADD has inherent limitations. Unlike traditional wet laboratory research, CADD primarily relies on computational processes, which depend heavily on the computer's processing power and the precise configuration of the experimental environment. It necessitates not only the effective application of software algorithms but also the abstraction of various specific experimental conditions into digital parameters [31]. Any deficiencies in these steps can adversely impact the accuracy of the results. Consequently, active supervision during calculations is essential when employing CADD.

Moreover, although AIDD is theoretically faster and more accurate than traditional CADD, the applications of AIDD in target inhibitors are limited. VS databases are often sourced from public chemical databases such as PubChem [81], ZINC [82], and ChEMBL [83], as well as experimental databases collected and established by individual research groups. Nevertheless, public databases lack comprehensive records of anti-caries drug activities, and inconsistencies in data quality and accessibility among research groups limit database choices for anti-caries drug design. This explains the limited application of AIDD methods in anti-caries drug design, as AIDD, particularly DL, requires high quality and quantity of data. The accuracy and predictive efficacy of these models are closely related to these factors. However, the potential of AIDD remains significant and represents a current hotspot in drug design. As dental caries-related databases continue to improve, we hypothesize that the AIDD methods will exhibit more significant value over time, with more and more attention being paid to dental caries research.

Property-based drug design (PBDD) is a holistic method that led to the evolution of small-molecule drug development. PBDD uses methods, including individual properties, property–activity relationships, sets of rules, composite metrics, multi-parameter optimization (MPO) scores, and AI/ML models, to optimize the pharmacological and safety profiles of drug molecules depending on the modulation of molecular and physicochemical properties [84]. The PBDD method holds promise as a direction for the development of more targeted and potent anti-virulence agents.

Currently, the targets for anti-caries drug development primarily focus on single or multiple cariogenic factors of *S. mutans*, including Gtfs, AgI/II, SrtA enzyme, the VicRK system, and SODs. In addition, biofilm-associated dihydrofolate reductase [85], oxidative stress-related nicotinamide adenine dinucleotide oxidase [86], and Dps-like peroxide resistance protein [87] play significant roles in *S. mutans*-induced carcinogenesis. Targeting other cariogenic bacteria, such as *Lactobacilli* [88] and *Actinomycetes* [89] for caries prevention, can also be considered a future direction for CADD-assisted anti-caries drug design.

In summary, the application of CADD in targeted anti-caries drug development has reached considerable maturity. However, its implementation in AIDD is constrained by a lack of high-quality databases, which poses a bottleneck. We support the establishment of unified experimental standards and the collection of openly accessible, high-quality data to build comprehensive databases for anti-caries drug development. This initiative aimed to pave the way for future applications of AIDD in the field of anti-caries drug design. In addition, CADD methods can be extended to target additional virulence factors and various cariogenic bacteria for anti-caries drug research and development.

## CRediT authorship contribution statement

**Zhongxin Chen:** Writing – original draft, Visualization, Formal analysis, Data curation, Conceptualization. **Xinyao Zhao:** Writing – original draft, Data curation. **Hanyu Zheng:** Writing – original draft, Data curation. **Yufei Wang:** Writing – review & editing, Supervision, Resources, Funding acquisition, Conceptualization. **Linglin Zhang:** Writing – review & editing, Supervision, Resources, Funding acquisition, Conceptualization.

## Declaration of competing interest

The authors declare that there are no conflicts of interest.
